# Study of slope length (*L*) extraction based on slope streamline and the comparison of method results

**DOI:** 10.1038/s41598-024-56696-w

**Published:** 2024-03-13

**Authors:** Jiayan Jiang, Mingliang Luo, Leichao Bai, Yunyun Sang, Shuo Yang, Hui Yang

**Affiliations:** School of Geographical Sciences, China West Normal University, Nanchong, 637009 China

**Keywords:** Slope streamline, Slope length extraction, Inverse terrain, DEM, Loess Plateau, Environmental sciences, Natural hazards, Ecology, Restoration ecology

## Abstract

Slope length is an important factor in soil erosion modeling, and the reasonable automatic extraction of slope length is of great significance in soil erosion research. However, previous studies have mainly focused on the regional scale, and how to effectively extract slope length at the slope scale deserves further research. In this study, a slope length extraction algorithm based on slope streamlines method (SSM) is proposed for the slope length extraction problem in geomorphology, and it is compared with three existing slope length calculation methods. The experimental results show that the new method can quickly calculate the length of slope streamlines, and the extracted slope lengths have better accuracy; the coefficients of determination demonstrates a better overall fitting effect of the four extraction methods, with coefficients of determination exceeding 0.7; this indicates that the use of SSM has similar accuracy and stability to other methods in calculating slope lengths. Among all the calculation methods, SSM has a better overall fitting effect for slope length calculation, and the obtained slope length value domain range is relatively small and concentrated in a small range, which expresses the slope length better.

## Introduction

In recent years, with the continuous development of computer science, research on geological analysis models and hydrological analysis has been characterized by high precision and multisource research^1–4^. Many soil erosion models currently use slope length as an important topographic factor, such as the Universal Soil Loss Equation (USLE)^5^, Revised Universal Soil Loss Equation (RUSLE)^6^, Wind Erosion Prediction System (WEPS)^7^, Water Erosion Prediction Project (WEPP)^8^, and European Soil Erosion Model (EUROSEM)^9^, and the accuracy of slope length calculation directly affects the accuracy of the geological analysis models^10,11^. Therefore, the identification and extraction of the slope length has become a hot topic for geographical research and has attracted widespread attention^9,12,13^.

Digital elevation models (DEMs) can represent and simulate the topographic morphology of the Earth’s surface digitally^14^. The inverse terrain DEM is important for terrain analysis as a form of transition analysis data constructed by a digital elevation matrix^15^. Many methods have been available to achieve automatic extraction of slope length based on DEMs^16–19^. However, because the DEM is a differential simulation of the ground surface, the design of its method is necessarily subject to various assumptions^20^. Different assumptions and premises can lead to different slope length calculation models and variability in the results. Although this has little impact on the visualization of terrain features and terrain classification, it has a significant impact on the geological analysis models that are calculated numerically^21–24^. Therefore, many scholars have carried out research on slope length calculation methods based on different models^25,26^. At present, the influence of slope length on soil erosion is mostly represented in soil erosion models in the form of slope length factors. Therefore, research by many scholars involves both the study of the actual slope length and the characterization of the slope length factors^27^. Foster and Wischmeier^28^ considered that the slope length of each segment can be considered as the accumulation of the slope length values of the upstream segments by performing a segmental analytical treatment of irregular slopes. Hickey et al.^29^ proposed calculating the cumulative downslope length based on the DEM in the grid system, which mainly calculated the maximum cumulative flow length from each grid cell to the starting point as the cumulative slope length from the grid to the top of the slope. Jin et al.^30^ focused on analyzing the influence of the water flow direction and the raster distance calculation on the slope length calculation based on the principle of the slope length method. Zhang et al.^31^ Proposed reflecting the slope length values of topographic factors by the distributed erosion slope length of watersheds, while the slope length was extracted by an iterative accumulation method. However, the previous calculation process used the edge-angle relationship, and often the slope at any point of the slope surface (point slope) was directly taken as a variable to solve the slope length based on the cosine relationship. The slope length in this algorithm is a cumulative quantity, while the slope is a local variable, and there is a contradiction between the two; at the same time, when the DEM of the grid for calculating the slope length becomes larger and the accuracy deteriorates, the error of the slope is very significant, which affects the calculation and expression of the slope length. Therefore, the calculation of slope length needs to consider a new algorithm.

Based on this, according to the problems and practical needs of the current research, this paper proposes a slope length extraction algorithm based on slope flow lines. By adopting the triangle side length relationship, it constructs the conversion relationship and solution method, and then extracts the slope length. It is also compared with three existing slope length calculation methods. The research results are intended to make useful attempts and new solution ideas for extracting slope length in the sense of geomorphology based on DEMs, which is of great significance to the research of topographic features.

## Materials and methods

### Study area

**Figure 1:**
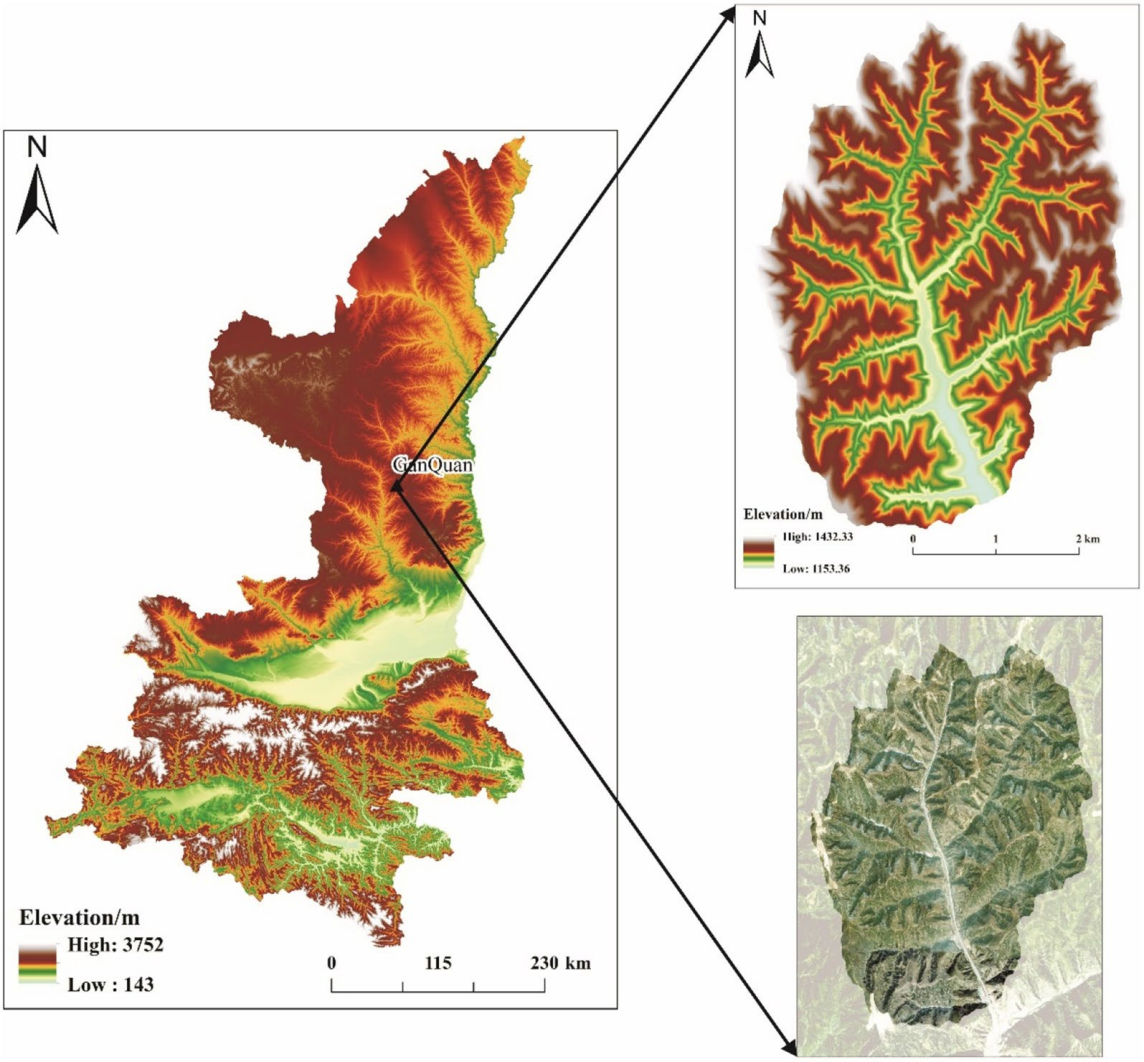
Map of the research area. The map was created using ArcGIS software, version 10.7 (Esri, Redlands, CA; www.esri.com).

In this research, the study was conducted in the Ganquan Loess hill ridge landform area of the Loess Plateau with steep terrain (Fig. 1). The Ganquan research area is located in Ganquan County, Shaanxi Province, China, in the west-central part of Yan’an city. It is situated in the middle reaches of the southeast Luohe River. Its center position is 109.543° E and 36.207° N. The area is mainly characterized by its unique geological structure and arid climatic conditions. The overall terrain slopes from northwest to southeast, with higher elevations in the northwest and lower elevations in the southeast. The density of gullies is 6.78 km/km^2^, the ground slope is high, and soil erosion is severe. The elevation in the region is between 1147 and 1458 m. Some gullies are obviously undercut, and in the accelerated cutting stage, the slope of gully edges reaches approximately 15–25°, but the slope between gullies can be as high as 50–80°. The geomorphological development stage is currently occurring, the degree of cut of regional gullies is high at approximately 0.47, and the depth of cut of gullies is approximately 1.045 m. The most widely distributed soil type in the region is loess, and loess-type soils are associated with poor moisture retention performance, high erosion and high drought susceptibility. The vegetation in the area is dominated by trees and shrubs, with some herbaceous meadows, crops, medicinal vegetation, etc. The forest coverage ratio is high, reaching 50.61%^32^.

### Research principle

In general, the slope length is the horizontal projected length of the slope surface, i.e., the maximum horizontal projected length of the trajectory between a given point against the flow and the start of the flow (also known as the source point)^33^. The general step of the solution is to obtain the slope length first, and then the projected length of any point on the slope, i.e., the slope length, is obtained based on the slope length, angle and other variables. If the slope length is regarded as the hypotenuse of a right triangle, then solving the slope length can be considered using the relationship between the sides of a right triangle or the side-angle relationship. Therefore, the key variables for solving the slope length are the length of the slope, the angle, or the difference in elevation from any point on the slope to the start of the streamline.

This algorithm uses the triangle side length relationship method to determine the slope length. The specific objective is to first calculate the length of the slope flow line, which can be calculated directly using the relevant software. Second, the difference in elevation (*∆H*) from any point on the slope to the starting point of the flow line is determined. For this purpose, it is necessary to trace back to the start of the flow line in the direction opposite the flow direction and then use the difference between the elevation of the start point and the elevation at any point on the flow line to obtain *∆H*. Finally, using the relationship between the hypotenuse (length of the flow direction) and *∆H* in the triangle, the slope length can be calculated. This method of calculating the slope length is highly adaptable for the terrain in the study region, and the principle is clear; this approach avoids the slope error problems caused by the low accuracy of the DEM. Therefore, we refer to this algorithm as the slope length extraction method based on slope streamlines (SSM). The algorithm calculation process is shown in Fig. 5.

### Experimental methods

#### Elevation difference (*∆H*) calculation

The key step in the algorithm is to calculate *∆H*, and the calculation steps are detailed below.

**Figure 2:**
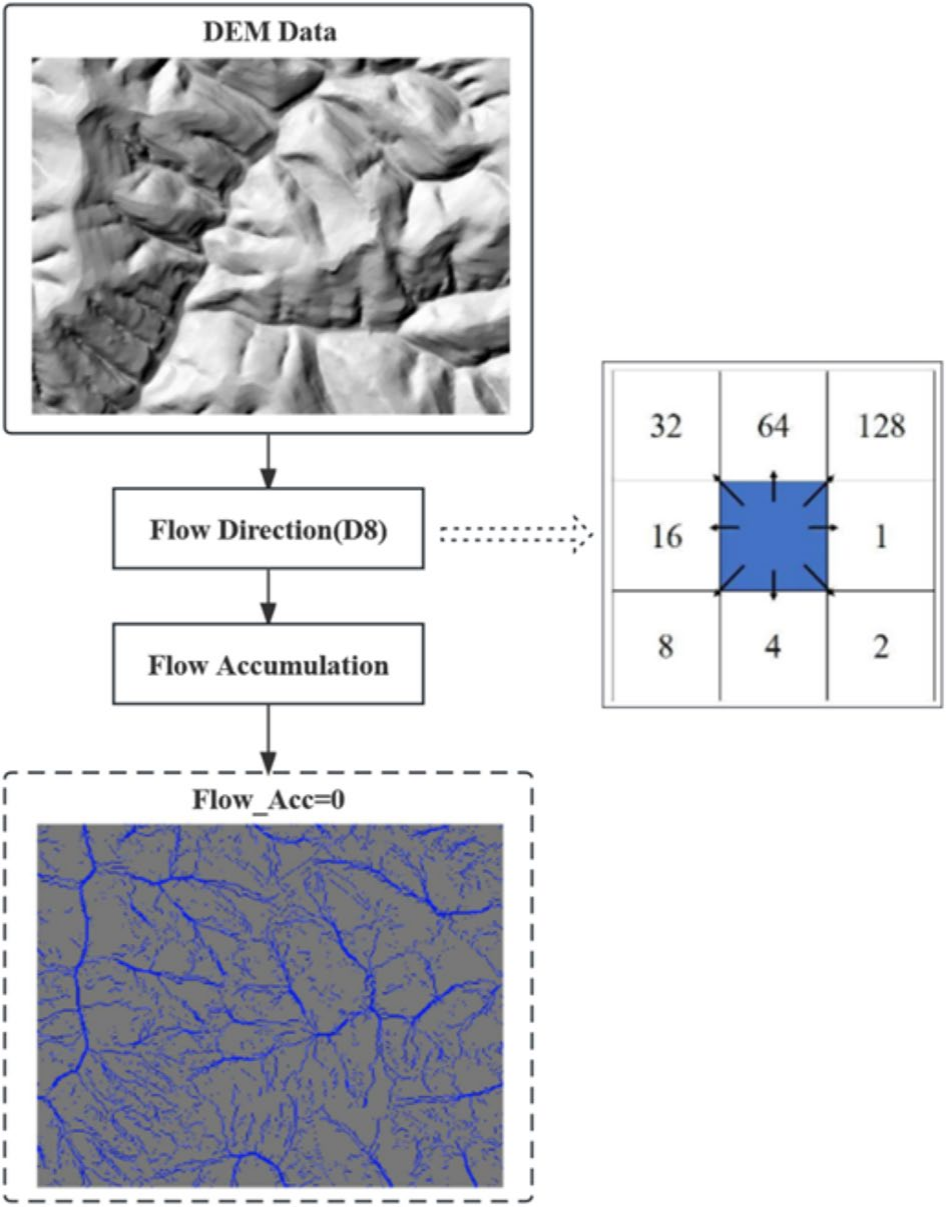
Streamline starting point layer extraction process.
- Flip the terrain and calculate the inverse terrain flow direction to invertdir. Point A is taken from the source line of the streamline, and according to the anti-terrain flow direction to invertdir, which grids flow to point A is determined, and the streamline is obtained. All the streamline starting point grids are traversed (watershed tool, where the flow direction is the anti-terrain flow direction, and the element tilt point is the flow direction starting point layer Sourcelay). The extraction process is shown in Fig. 3.

- The D8 algorithm is used to calculate the flow accumulation in the sample area according to the flow direction and flow accumulation steps, and the grid with the flow accumulation of 0 is calculated, which is named Sourcelay, that is, the streamline starting layer. The extraction process is shown in Fig. 2.

**Figure 3:**
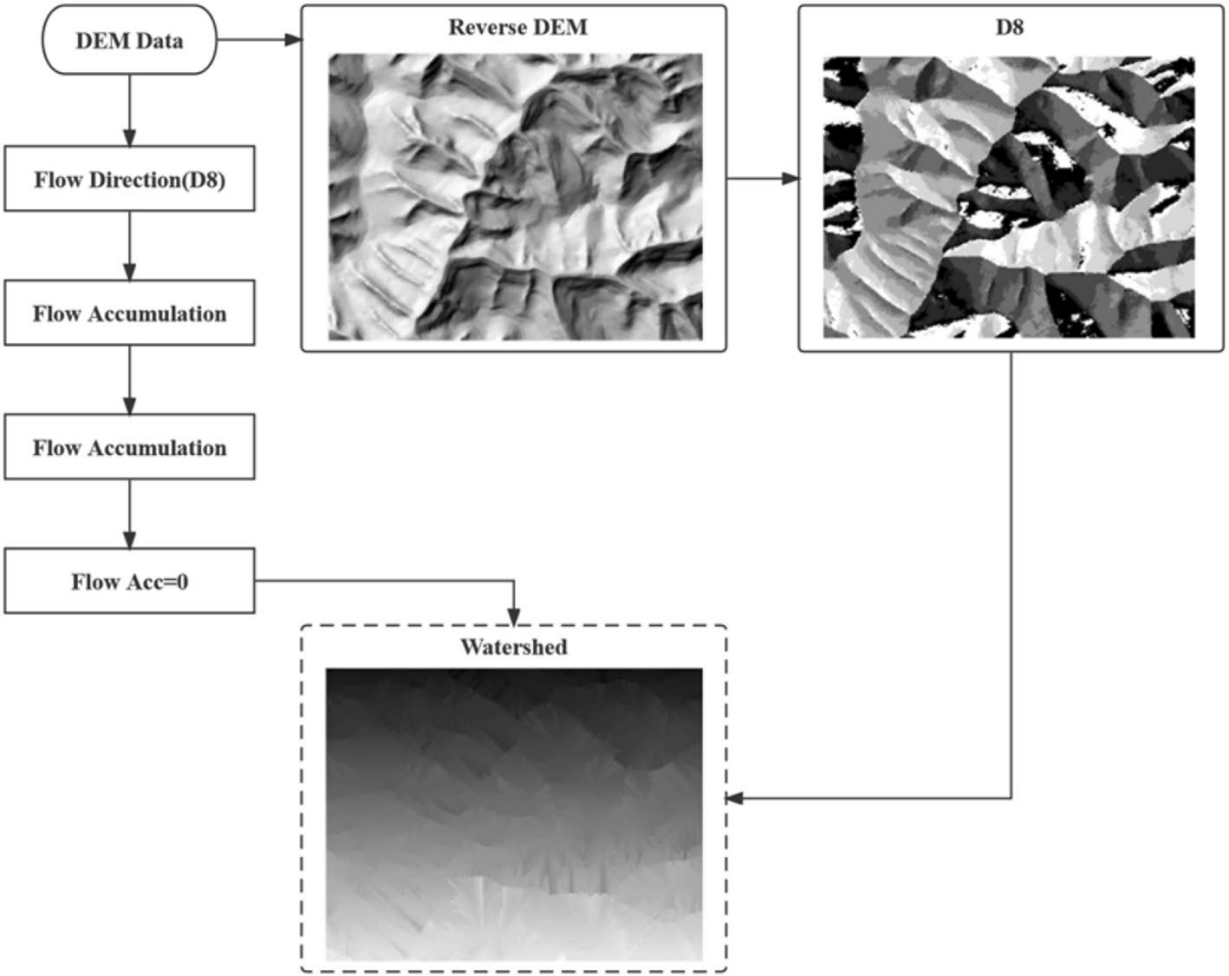
Watershed tool extraction process.
- According to the streamline partition, the elevation difference *∆H* (Fig. 4), between any point on the streamline and the starting point of the corresponding streamline is calculated (Zonal statistical tool, streamline identifies a specific area) (Fig. 5).

**Figure 4:**
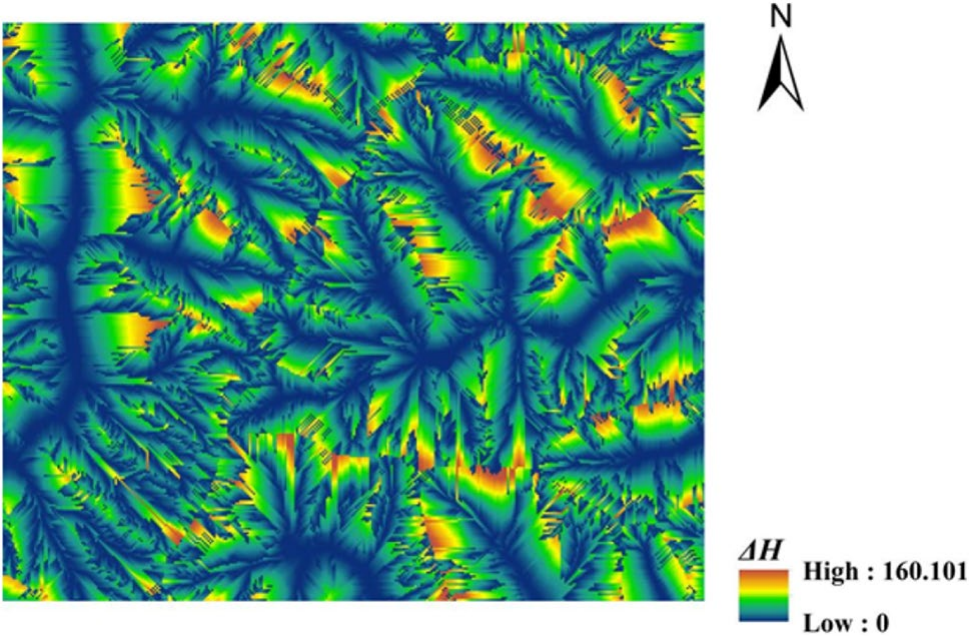
The elevation difference *∆H* from any point on the streamline to the corresponding streamline starting point.

**Figure 5:**
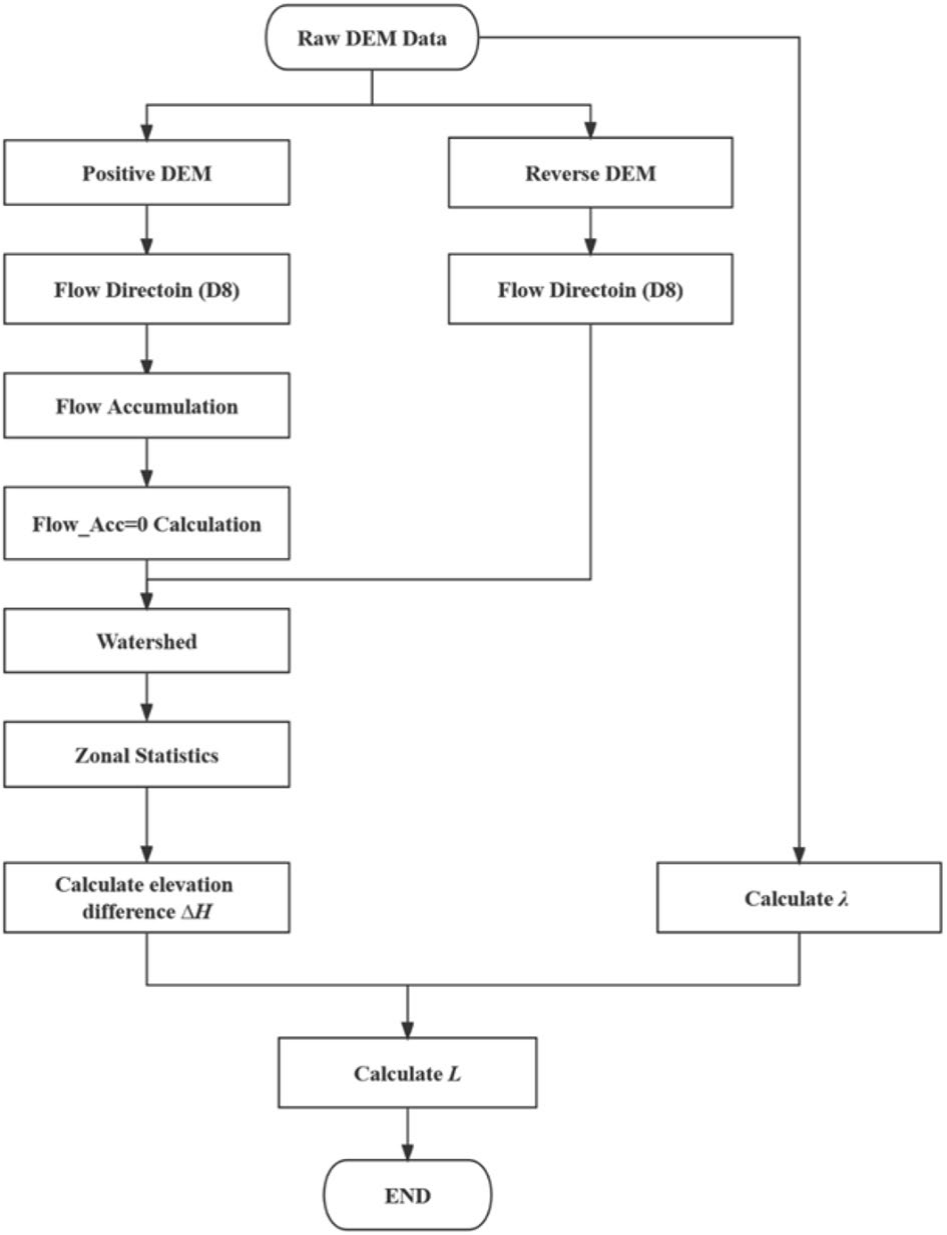
Flow chart of slope length (*L*) calculation by the SSM.

#### Calculation of flow line surface length (*λ*)

Flow path length is calculated using the SAGA GIS software and results in a streamline surface length (*λ*). Based on the D8 algorithm^34^, the average flow path length from the seed point to each cell is accurately calculated to analyze the flow of water over the terrain surface^35^.

#### Slope length (*L*) calculation

The slope length is the horizontal projection length of the slope surface, i.e., the maximum horizontal projection length of the trajectory between a given point against the flow and the start of the flow (also known as the source point). Therefore, based on the elevation difference obtained from the calculation and the length of the flow line on the slope surface, *L*. The formula is as follows (Eq. 1).

1 $$L=\sqrt{{\lambda }^{2}-{\Delta H}^{2}}$$

In the equation, *L* represents the slope length. *λ* represents the length of the water flow path on the slope surface, which is the same as *L*. *∆H* is the elevation difference, that is, the elevation difference between the previous point in the streamline and the corresponding streamline starting point.

#### Experimental comparison

In the entire research area, the DEM pixel size is 5 m × 5 m, there are 1552 × 1419 grids in this area, and the file size is 8.40 MB. The SSM-calculated slope length was compared with the SL_zh_, SL_laya_ (slope length calculation in SAGA GIS) and SL_fu_ results, and the corresponding plots, statistical results and accuracy of the calculations of slope length were analyzed.

The SL_zh_ method is mainly used to extract slope length by using the extraction method proposed by Zhang et al.^36^. In this method, the slope length is determined by analyzing changes in the terrain. Currently, it is implemented in the NET environment using C#. The application is designed to be simple and user friendly.

The SL_laya_ method (V. Olaya (c)) is provided in SAGA GIS^35^, and we use SAGA GIS to calculate slope length. SAGA GIS is a free and open-source geographic information system software widely used for terrain analysis and geomorphological research. The algorithm is based on benchmark elevation data and calculates slope length using input parameters.

The SL_fu_ method was created by Fu et al.^37^ The development tool used to run the application is Visual Studio 2010, with the algorithm implemented in C^++^ and the interface in C#. This application takes user convenience into consideration, featuring a friendly and clear interface and allowing the direct viewing of calculation results within the software.

### Statistical analysis method

#### Frequency and cumulative frequency curves

- Frequency refers to the ratio of the occurrence times of certain data to the total number of data, usually expressed as a percentage, as shown in Eq. (2).

2 $${f}_{i}=100\times \frac{{n}_{i}}{\sum_{i=1}^{j}{n}_{i}}$$

where *f*_*i*_ denotes the frequency corresponding to value *i*, *n*_*i*_ denotes the number of occurrences of value *i*, and $\sum_{i=1}^{j}{n}_{i}$ denotes the total number of data.

- Cumulative frequency refers to the sum from the lowest frequency of the variable value to the maximum frequency, and the final cumulative value is 100%, as shown in Eq. (3).
  3 $${F}_{i}=\sum_{i=1}^{j}{f}_{i}$$
  where *F*_*i*_ denotes the cumulative frequency corresponding to value *i*, and $\sum_{i=1}^{j}{f}_{i}$ is the sum from the first frequency accumulated to the i-th frequency.

In this study, the slope length values calculated by the four different slope length methods were used as the X-axis, and the percentile values were used as the Y-axis to plot the frequency curves. The cumulative frequency curves are plotted by taking the slope length values calculated by the four different slope length methods as the X-axis and the percentile values as the Y-axis.

#### XY scatter plot

Taking one slope length calculation method as the benchmark method and another slope length method as the method for comparison, with the X-axis indicating the benchmark method and the Y-axis indicating the method of comparison, the XY scatter plot of the two methods can be drawn based on the two-dimensional spatial coordinates. The XY scatter diagram is a kind of statistical diagram that shows the relationship between the values in the data series in a graphical way and can compare the similarity and correlation of the two methods more intuitively, and its distribution relationship is shown in Table 1. Meanwhile, for the equation Y = ax + b fitted on the XY scatter plot, the difference between the two methods increases as the deviation from 0 or 1 increases. Linear regression analysis was performed based on the XY scatter plot with the coefficient of determination, R^2^, as the discriminant value, and when it is closer to 1, it indicates that the degree of similarity of the fit is higher^38,39^.

**Table 1:** XY scatter distribution of different slope length methods.

Compare	Benchmark			
	SL_zh_(x)	SL_laya_(x)	SL_fu_(x)	SSM(x)
SL_zh_(y)				
SL_laya_ (y)				
SL_fu_ (y)				
SSM(y)				

#### Spatialized relative difference coefficients

In this study, we improve the relative difference coefficients that were previously expressed in numerical values. Unlike the conventional method, the spatialized relative difference coefficient approach accounts for the influence of the neighborhood scale on the similarity of the slope length values. Specifically, we extend the original relative difference coefficient method based on single-point slope length values to compare the slope length values within a local area centered around a specified neighborhood and thus express the spatial similarity at different scales. The spatialized relative difference coefficients are used to quantify the differences between the different slope length methods, and the results generated with the four slope length calculation methods, namely, the SL_zh_, SL_laya_, and SL_fu_ methods and SSM, are interactively compared, with one selected as the benchmark method and the others as the compared methods^38,39^. The relative difference coefficient α can be defined as shown in (Eq. 4):

4 $$\alpha =1-\frac{{\sum }_{i=1}^{n}{\left({A}_{comp}^{i}-{A}_{base}^{i}\right)}^{2}}{{\sum_{i=1}^{n}({A}_{comp}^{i}-\overline{{A }_{base}^{i}})}^{2}}$$

where ${A}_{base}^{i}$ is the slope length value of the benchmark method for cell *i*, $\overline{{A }_{base}^{i}}$ is the average value of the slope length obtained with the benchmark algorithm in a certain spatial range, and ${A}_{comp}^{i}$ is the slope length value of the compared algorithm at the corresponding position. The relative difference coefficient $\alpha$ represents the total deviation degree between the compared and benchmark algorithms. When $\alpha =1$, the calculation results of the two slope length methods are identical. The smaller α is, the greater the difference between the slope length calculation methods. When $\alpha <0$, the two slope length calculation results are not comparable. Considering the relative difference coefficient α values of different results together, the degree of difference between the compared algorithms and the benchmark algorithm can be comprehensively evaluated. In turn, the most suitable slope length calculation method for terrain slope length studies in the region can be determined.

### Ethical approval

All authors have read, understood, and have complied as applicable with the statement on “Ethical responsibilities of Authors” as found in the Instructions for Authors and are aware that with minor exceptions, no changes can be made to authorship once the paper is submitted.

## Results

### Analysis of terrain characteristics

**Figure 6:**
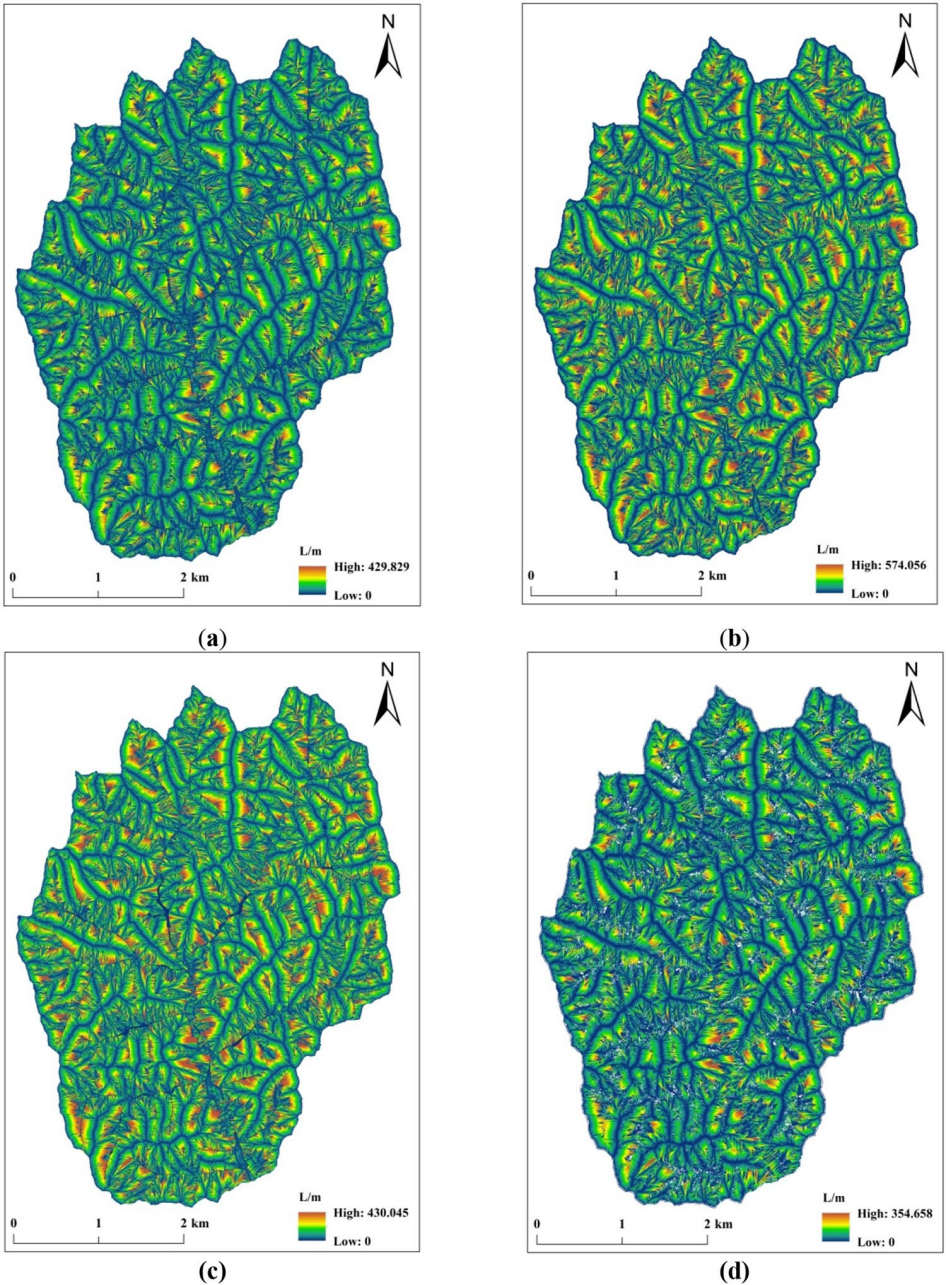
Extraction results of slope length based on different methods. (**a**) Extraction results of the slope length calculated by the SL_zh_ method. (**b**) Extraction results of the slope length calculated by the SL_laya_ method. (**c**) Extraction results of the slope length calculated by the SL_fu_ method. (**d**) Extraction results of the slope length calculated by the SSM.

The topographical property index is the most effective parameter index to express and study the surface morphology, and it is also the most intuitive characterization to judge the accuracy of the DEM data. The results of the four slope length calculation methods discussed in this study are shown in Fig. 6. The statistical results of the characteristic values of the terrain attribute indexes are shown in Table 2. As seen from Table 2, the slope length values calculated by the SSM and the other three methods differ when the DEM data are the same and the slope length calculation methods are different, which is related to the different ways of calculating the flow direction during the slope length calculation. Meanwhile, the maximum value, average value and standard deviation of slope length calculated by the SSM are smaller than those of the other three methods, indicating to a certain extent that the fluctuation range of the slope length calculated by the SSM is smaller than that calculated by the other three methods. In summary, by comparing the statistical results of the eigenvalues of the four slope length calculation methods, this study concludes that although there are some calculation results of the SSM where there are differences that exist when it is compared with the other three methods, the SSM stability advantage shows that it can reliably reflect the morphological characteristics of the ground surface.

**Table 2:** Distribution value statistics of the DEM topographic factor features.

Method	Topographic statistics	Max	Min	Mean	Std
SL_zh_	Elevation/m	1432.33	1153.36	1297.44	55.8
	slope length/m	429.83	0	49.89	45.32
SL_laya_	Elevation/m	1432.33	1153.36	1297.44	55.8
	slope length/m	574.06	0	48.71	50.96
SL_fu_	Elevation/m	1432.33	1153.36	1297.44	55.8
	slope length/m	430.05	0	50.85	46.96
SSM	Elevation/m	1432.33	1153.36	1297.44	55.8
	slope length/m	354.66	0	39.01	37.72

### Analysis of regulation in the slope length distribution

**Figure 7:**
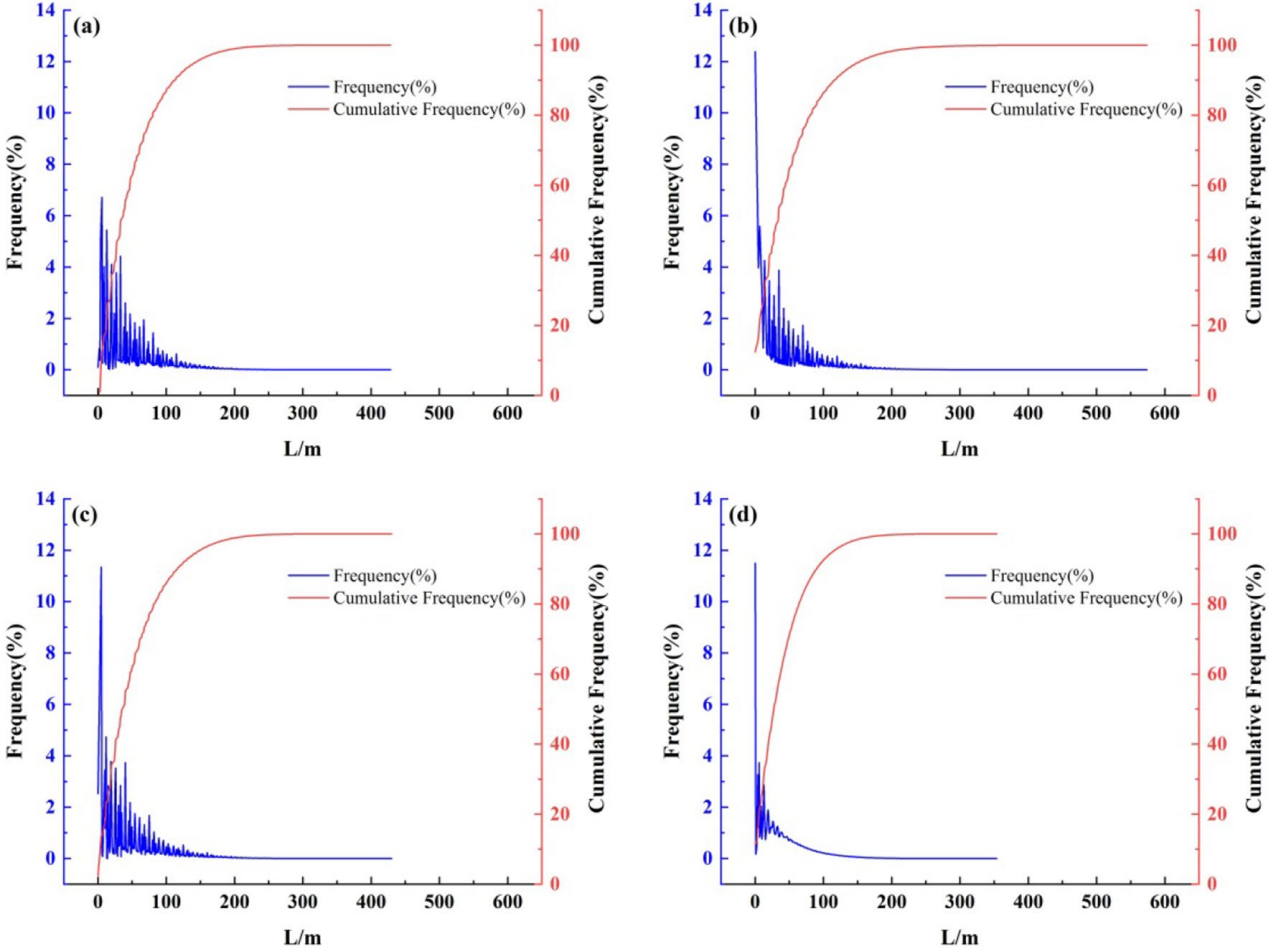
Frequency and cumulative frequency curves generated by different slope length calculation methods. (**a**) Slope length frequency and cumulative frequency curves generated by the SL_zh_ method. (**b**) Slope length frequency and cumulative frequency curves generated by the SL_laya_ method (**c**) Slope length frequency and cumulative frequency curves generated by the SL_fu_ method (**d**) Slope length frequency and cumulative frequency curve generated by the SSM.

Frequency statistical analysis is used primarily to reflect the distribution pattern of the slope length values and record the number of grids in different ranges of slope length values on the whole slope length grating surface. In this study, the frequency of slope length results of different calculation methods are compared and analyzed to determine the differences in the results from the four calculation methods (Fig. 7). In the range of 0–150 m, the SL_zh_, SL_laya_ and SL_fu_ methods all show a large frequency with the largest percentage of area, drastic changes in slope length, and high instability, indicating that the fluctuation range of slope length calculated by the three methods is larger in this interval, while when the slope length is larger than 150 m, the slope length frequency curve changes regularly with a smaller fluctuation range. In contrast, for the SSM, the slope length frequency curve varies regularly when the slope length value is greater than 50 m, indicating that the range of fluctuation is small when the slope length value is greater than 50 m. On the other hand, when the slope length is less than 50 m, the area share is the largest, the change in the slope length is drastic, and the instability is strong, which indicates that the range of the fluctuation of the slope length is large in this interval. The areas with cumulative frequencies of SL_zh_, SL_laya_, SL_fu_ and SSM < 50 m accounted for 62.8%, 64.8%, 62.0% and 71.0%, respectively, of which SSM accounted for the largest proportion. When the slope length value is < 100 m or less, the cumulative frequency ratio is SSM (92.5%) > SL_zh_ (86.9%) > SL_laya_ (86.4%) > SL_fu_ (86.0%), and the cumulative frequency area ratio of the areas with slope length values < 150 m for SL_zh_, SL_laya_, SL_fu_, and SSM are 96.2%, 96.0%, 96.0%, 98.4%, respectively, with SSM having the largest ratio, 96.0%, and 98.4%, with SSM accounting for the largest proportion. In general, the cumulative frequency distribution curves obtained by the SL_zh_, SL_laya_, and SL_fu_ methods and the SSM method in this study present similar overall trends, all of which show a gradually increasing and gradually flattening pattern. This indicates that the information on the spatial distribution of slope length measured by the four DEM datasets is basically the same, while the differences are mainly reflected in the distribution of flow.

### Comparison of the correlation degree between different methods

The correlation analysis and performance index of slope length extracted by different methods are shown below. According to the results in Tables 3 and 4, it can be found that there is a certain correlation between slope length extracted by different methods, and the coefficient of determination of the linear fitting results between the four methods is above 0.7, indicating that the model can explain the variation of variables well. At the same time, the minimum MAE is 6.39, the minimum RMSE is 18.07, and the maximum VAF is 81.05. These indicators also reflect the prediction ability of the model. When the slope length result calculated by the SL_zh_ method is used as the X-axis, it results in R^2^ values of 0.81, 0.79 and 0.74, with the results of the SL_laya_ and SL_fu_ methods and the SSM for slope length calculation in this study, respectively. The difference between the three calculation methods is not significant, which indicates the reliability of the accuracy of the proposed method applied to slope length extraction in this study and verifies the applicability of the method in this region. The correlation with the SL_zh_ method is the highest when the slope length calculated by the SL_laya_ method is the X-axis, with an R^2^ of 0.81, and the correlation with the SL_fu_ method and the SSM is lower than the former, with correlation coefficients of 0.71 and 0.72, respectively. When the slope length results calculated by SL_fu_ were used as the X-axis, the methods with the highest to lowest correlation with their coefficients of determination R^2^ were SL_zh_, SSM, and SL_laya_ methods, with values of 0.79, 0.77, and 0.71, respectively; SL_fu_ had the highest correlation with its slope length results calculated by SSM as the X-axis (R^2^ = 0.77), SL_zh_ had a slightly lower correlation than the former (R^2^ = 0.75), and SL_laya_ had the lowest correlation among the three compared methods (R^2^ = 0.72), the MAE (10.24) and RMSE (22.73) of SSM(x) and SL_fu_ were smaller, while the VAF was larger (77.04%). In summary, the SSM proposed in this study is reliable and applicable in terms of accuracy when applied to slope length extraction. In particular, the correlation coefficient of the SSM method is the highest with SL_fu_ when the result is calculated for the X-axis for SL_fu_, which further verifies that these two algorithms are more similar to each other.

**Table 3:** Correlation analysis of different slope length calculation methods.

Compared to	Benchmark			
	SL_zh_(x)	SL_laya_(x)	SL_fu_(x)	SSM(x)
SL_zh_(y)		y = 0.8064x + 10.874 R^2^ = 0.81	y = 0.8588x + 6.3567 R^2^ = 0.79	y = 1.0483x + 10.042 R^2^ = 0.75
SL_laya_(y)	y = 1.0051x−1.4962 R^2^ = 0.81		y = 0.909x + 2.5111 R^2^ = 0.71	y = 1.1512x + 4.7802 R^2^ = 0.72
SL_fu_(y)	y = 0.9242x + 4.8549 R^2^ = 0.79	y = 7848x + 12.936 R^2^ = 0.71		y = 1.1039x + 8.8547 R^2^ = 0.77
SSM(y)	y = 7132x + 2.7018 R^2^ = 0.75	y = 0.6285x + 7.8041 R^2^ = 0.72	y = 0.6979x + 2.7937 R^2^ = 0.77

**Table 4:** Performance index of different slope length calculation methods.

Compared to	Benchmark	Index		
		MAE	RMSE	VAF (%)
SL_zh_(y)	SSM(x)	10.31	22.97	74.77
	SL_fu_(x)	6.64	20.77	79.37
	SL_laya_(x)	8.06	19.90	81.05
SL_laya_(y)	SSM(x)	11.77	26.84	72.35
	SL_fu_(x)	7.89	27.33	71.34
	SL_zh_(x)	6.39	22.22	81.05
SL_fu_(y)	SSM(x)	10.24	22.73	77.04
	SL_laya_(x)	10.59	25.39	71.34
	SL_zh_(x)	7.31	21.55	79.37
SSM(y)	SL_fu_(x)	9.98	18.07	77.04
	SL_laya_(x)	11.21	19.83	72.35
	SL_zh_(x)	10.45	18.95	74.77

### Relative spatial differences in the correlation coefficient analysis among the different slope length calculation methods

**Figure 8:**
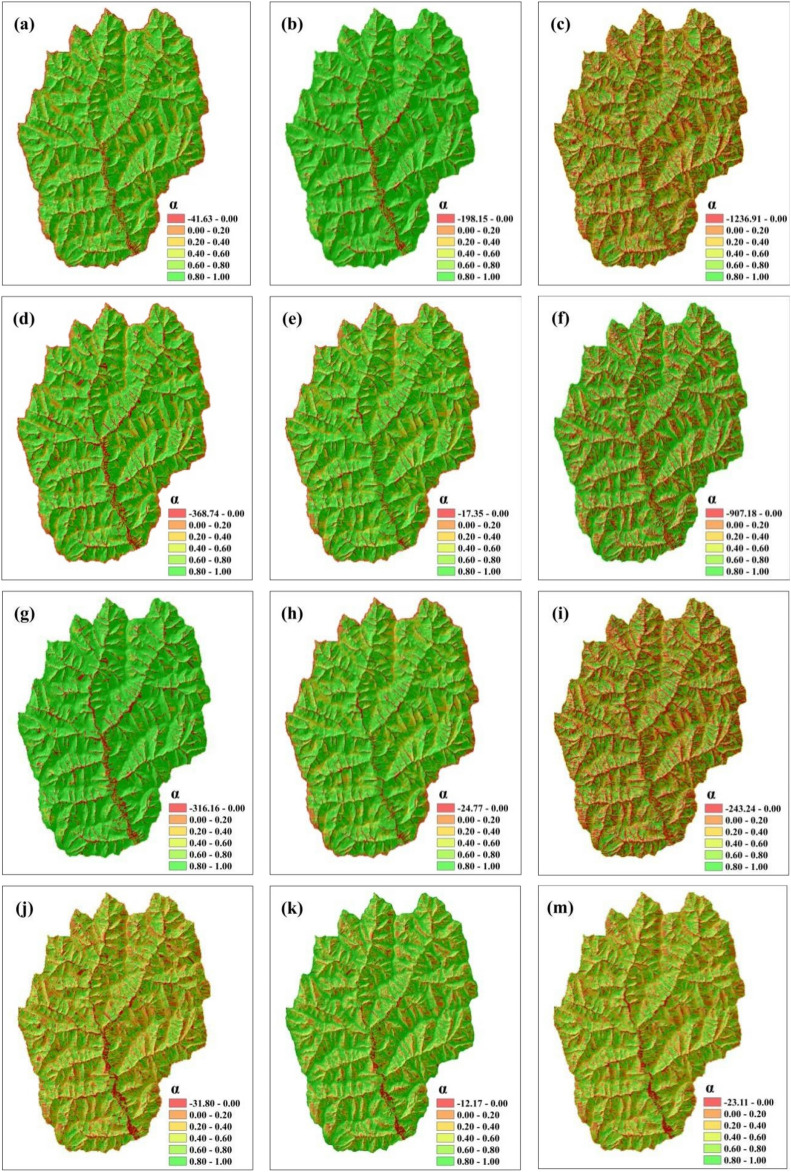
Spatial relative difference coefficients calculated by different slope length calculation methods. (**a**)–(**c**) the spatial relative difference coefficients generated by the SL_zh_ method as the base algorithm and the SL_laya_, SL_fu_ and SSM method as the comparison algorithms in turn. (**d**)–(**f**) The spatial relative difference coefficient maps generated by the SL_laya_ method as the benchmark algorithm and the SL_zh_ and SL_fu_ methods and the SSM as the comparison algorithms, respectively. (**g**)–(**i**) The spatial relative difference coefficient maps generated by the SL_fu_ method as the benchmark algorithm and the SL_zh_, SL_laya_ and SSM methods as the comparison algorithms, respectively. (**j**)–(**m**) The spatial relative difference coefficient maps generated by the SSM as the benchmark algorithm and SL_zh_, SL_laya_, and SL_fu_ methods as the comparison algorithms, respectively.

By extending the relative differences of the correlation coefficients of the methods for the single-point slope length calculation values, the slope length values at different scales can be compared, and the spatial similarity can be quantified. The slope length results generated by the four methods are compared interactively to finally obtain a correlation coefficient map reflecting the relative spatial differences (Fig. 8). As seen from the figure, when the slope length results calculated by the SL_zh_ method are used as the benchmark and the SSM is used as the comparison algorithm, we find that its area with α ≥ 0.8 accounts for 18.22% of the total area, which is mainly distributed in the slope part of the hillside, indicating that the spatial similarity of the slope length results between the SL_zh_ method and the SSM in the slope part of the hillside is high, while 25.91% of the area with α ≤ 0 is mainly distributed in part of the ridge and the bottom part of the gully, indicating that in this area the two methods are not comparable. When the slope length results calculated by the SL_laya_ method are used as the benchmark, the part of the SSM with α ≥ 0.8 accounts for 46.05% of the total, which is distributed mainly at the slope surface and part of the ridge, while the area with α ≤ 0 accounts for 20.54% of the total, which is mainly distributed at the bottom part of the gully. When taking the slope length results calculated by the SL_fu_ method as the benchmark, the part of the SSM with α ≥ 0.8 only accounts for 17.10% of the total, and only a small part of it is distributed on the slope surface, indicating that the slope length results calculated by the SL_fu_ method and the SSM in the slope surface part have high similarity, while the area with α ≤ 0 or more accounts for 28.22% of the total. When the slope length results calculated by the SSM are taken as the benchmark and the SL_laya_ method is used as the comparison algorithm, the area with α ≥ 0.8 is 46.07%, which is the highest value, indicating that the two methods have high similarity at the slope face and part of the ridge, while when α ≤ 0, the SL_laya_ method only accounts for 4.12% of the total, which is mainly distributed at the bottom part of the gully, indicating that the two methods are not comparable at the bottom part of the gully. In summary, the spatial similarity between the results obtained by different methods is high in regions with similar topographic features. Thus, in these regions, the slope length results obtained by various methods are relatively consistent and display strong spatial consistency. On the other hand, in some regions with complex topography, there may be large differences between the results obtained by different methods. This indicates that when the terrain features are complex and varied, the results of various calculation methods are more variable and may be affected by factors such as terrain undulations and slope changes.

## Discussion

As an important topographic parameter in studies such as for soil erosion purposes, the slope length mainly regulates hydrological processes by influencing runoff aggregation at different topographic sites, thus affecting the size and rate of soil erosion. In recent years, the slope length algorithm has been widely used in numerical simulations and field monitoring by scholars at home and abroad and has been widely verified and recognized^3,40^. The results of this study show, to a certain extent, that the slope length values calculated by the SSM and the other three methods examined in this study are somewhat different, and the mean and maximum values of the extracted slope lengths via the SSM are smaller than those extracted by the other methods. The main reason for the discrepancy between the results of slope length extraction by the SSM and those obtained by the SL_zh_, SL_laya_ and SL_fu_ methods is due to the great difference in slope length assignment by the flow direction algorithm. In the SL_zh_ calculation method, the D8 direction algorithm was mainly selected to calculate the cumulative slope length independently in eight directions, accounting for the effect of trench truncation on the slope length^36^. The maximum slope drop method is used in the SL_fu_ calculation method, i.e., the best representative value of the grid slope is the maximum value of the slope in eight directions around the 3 × 3 window, i.e., maintaining the flow direction in line with the maximum slope drop value^37^. For the SSM method proposed in this study, although the single flow direction algorithm is also considered in the selection of the flow direction, the SSM method does not involve the calculation of the truncation factor, i.e., it does not account for the influence of the truncation factor on the extraction of the slope length value thus leading to the discrepancy. At the same time, when the terrain is highly undulating and the local changes are drastic, the single-flow direction algorithm is easily affected by such changes, thus generating errors. This study found that for the SSM calculation, when the slope length value is greater than 50 m, its slope length frequency curve regularly changes. When the slope length is less than 50 m, the slope length frequency curve changes sharply, and instability is enhanced. Cheng Zheng^41^ extracted the erosional slope length and found that the frequency curve of the slope length changes smoothly in the multiflow direction algorithm, while the single-flow direction algorithm is easily affected by the change in slope, resulting in a sharp change in the slope length and a strong instability. This result is consistent with the dramatic frequency variation when the slope length is less than 50 m, considering the single-flow direction algorithm for slope length extraction in this study. Therefore, it is very important to choose the correct calculation method when calculating slope length. Only when data characteristics and application scenarios are fully considered can accurate and reliable results be obtained.

Relevant studies have shown that there is a linear correlation between the DEM terrain description error and the DEM spatial resolution and ground roughness, and the resolution is an important factor affecting the morphological feature expression with a DEM and index calculation results^42,43^. In this study, the size of the grid used in the DEM is 5 m × 5 m. Therefore, limited by the data structure of the grid DEM, the calculation results have certain uncertainties. The study area is in the Loess Plateau region, with discontinuous topographic features and complex topographical elements; therefore, any results are significantly affected by the DEM resolution. Moreover, the recognition of local elevation points in the sample area and the processing method of the DEM data have important effects on slope length extraction. Therefore, despite the potential accuracy of the new *L* calculation method for slope length extraction, extensive testing in various small watersheds is still necessary to ensure its reliable application.

To evaluate the effectiveness of the SSM in slope length extraction, the slope length data of the SSM, SL_zh_, SL_laya_ and SL_fu_ methods are analyzed. Their numerical and spatial results are compared. First, by calculating the correlation coefficients among the methods, it can be found that the SSM has a higher correlation with the SL_fu_ method but a lower correlation with the SL_zh_ and SL_laya_ methods. This result indicates that the algorithm ideas and implementation process of the SSM and SL_fu_ method are similar. Meanwhile, there are similarities in the manner of defining the local elevation starting point to the end point of the runoff. However, the SL_zh_ and SL_laya_ methods adopt different ideas and methods, so the prediction results are slightly different from those of the other two algorithms. Second, compared with the SL_zh_ and SL_fu_ methods, when slope length results calculated by the SL_laya_ method were used as the basis for comparison, the spatial relative difference coefficient of the SSM accounted for 46.05% of the total above 0.8, which was mainly distributed in the hillsides and part of the ridge. It shows that the two methods have a high spatial similarity in these regions. This performance is also reflected in the higher distribution of relative difference coefficients of the SL_laya_ method when compared with other methods when the SSM is used as the base algorithm for comparison, which is also mainly shown at the slope face and part of the ridge. Overall, from the results of the spatial relative difference coefficients and the correlation coefficients, the different slope length calculation methods have a high spatial similarity in areas with similar topographic features. Li et al.^44^ studied the slope length factors of the Tibetan Plateau and found that the geomorphology of the Tibetan Plateau (especially in the marginal areas) is in a very young state. This tectonic geomorphic phenomenon causes the three topographic indexes of the plateau margin to be larger than the spatial pattern phenomenon inside the plateau, namely, the slope, slope length (*L*) and slope factor (*S*). From a macroscale perspective, it is found that the slope of the Qinghai-Tibet Plateau is steeper on all sides, and has a lesser slope in the middle. By comparing the spatial relative difference coefficients it is found in this study that the spatial similarity of the results obtained by different methods is higher in regions with similar topographic features, which also have gentler characteristics within these regions. The actual geomorphic form is usually three-dimensional, and its relief varies greatly in different drainage basins. For example, truncation errors caused by the extraction of terraces and drainage ditches will affect extraction accuracy, so this method still needs to be repeatedly tested in gully areas with complex morphological structures. Studies have shown that when channel truncation is considered, the accuracy of slope length extraction is greatly improved^45^. Therefore, a larger study area can be selected in a later stage to further explore the influence of the truncation factor and the DEM accuracy on the extraction effect of the slope length.

## Conclusion

In this study, by analyzing the spatial characteristics of the terrain, a slope length extraction method based on the length of slope flow lines considering spatial characteristics is proposed. The main conclusions are as follows:

1. The slope length values calculated by the SSM and those calculated by the other three methods are somewhat different, and the mean and maximum values of the extracted slope lengths are smaller than those extracted by the other methods. Moreover, the cumulative frequency distribution curve obtained by the SSM presents an overall trend similar to that of the other three methods. This indicates that the spatial distributions of the slope length measured by the four DEMs are basically the same, while the differences are mainly reflected in the flow distribution.
2. The coefficients of determination of the linear fitting results among the four methods are all above 0.7, demonstrating the reliability of the SSM. The correlation coefficient of the SSM is the highest with respect to SL_fu_ when the SL_fu_ calculation result is taken as the X-axis.
3. In regions with similar topographic features, the spatial similarity between the results obtained by different methods is high. However, in regions with complex terrains, there are differences between the results obtained by different methods. This indicates that when the terrain features are complex and diverse, the results of various calculation methods are more different, which may be affected by factors such as terrain undulation and slope change.

In summary, the slope length extracted by the slope streamline method (SSM) is in line with geomorphological knowledge and has certain geomorphological significance; this approach is useful for extracting the slope length in the geomorphologic sense.

## Data Availability

The datasets used and/or analysed during the current study available from the corresponding author on reasonable request.
